# A Key Role of Dendritic Cells in Probiotic Functionality

**DOI:** 10.1371/journal.pone.0000313

**Published:** 2007-03-21

**Authors:** Benoit Foligne, Georgia Zoumpopoulou, Joelle Dewulf, Amena Ben Younes, Fabrice Chareyre, Jean-Claude Sirard, Bruno Pot, Corinne Grangette

**Affiliations:** 1 Laboratoire de Bactéries Lactiques et Immunité des Muqueuses, Institut Pasteur de Lille - Institut de Biologie de Lille, Lille, France; 2 Institut National de la Santé et de la Recherche Médicale (INSERM)U801, Institut Pasteur de Lille - Institut de Biologie de Lille, Lille, France; 3 IFR142, Institut Pasteur de Lille - Institut de Biologie de Lille, Lille, France; 4 Institut National de la Santé et de la Recherche Médicale (INSERM) U674, Fondation Jean Dausset/CEPH, Paris, France; New York University School of Medicine, United States of America

## Abstract

**Background:**

Disruption of the intestinal homeostasis and tolerance towards the resident microbiota is a major mechanism involved in the development of inflammatory bowel disease. While some bacteria are inducers of disease, others, known as probiotics, are able to reduce inflammation. Because dendritic cells (DCs) play a central role in regulating immune responses and in inducing tolerance, we investigated their role in the anti-inflammatory potential of probiotic lactic acid bacteria.

**Methodology/Principal Findings:**

Selected LAB strains, while efficiently taken up by DCs *in vitro*, induced a partial maturation of the cells. Transfer of probiotic-treated DCs conferred protection against 2, 4, 6-trinitrobenzenesulfonic acid (TNBS)-induced colitis. Protection was associated with a reduction of inflammatory scores and colonic expression of pro-inflammatory genes, while a high local expression of the immunoregulatory enzyme indolamine 2, 3 dioxgenase (IDO) was observed. The preventive effect of probiotic-pulsed DCs required not only MyD88-, TLR2- and NOD2-dependent signaling but also the induction of CD4+ CD25+ regulatory cells in an IL-10-independent pathway.

**Conclusions/Significance:**

Altogether, these results suggest that selected probiotics can stimulate DC regulatory functions by targeting specific pattern-recognition receptors and pathways. The results not only emphasize the role of DCs in probiotic immune interactions, but indicate a possible role in immune-intervention therapy for IBD.

## Introduction

Dendritic cells (DCs) are antigen presenting cells (APC) that play a central role in orchestrating immune responses to self and foreign antigens. Contact with antigens or inflammatory stimuli, can induce the maturation of DCs, accompanied by functional and phenotypic changes. In their mature state, the DCs are primed to activate T cells towards a Th1/Th2 polarization. In the absence of inflammation, the DCs remain in an immature state, leading to either the deletion of effector T cells or the generation of regulatory T cells (Treg) [1]. Recent data highlight the critical role of naturally occurring CD4+CD25+ Treg cells in the control of normal immune homeostasis [2]. Little is known, however, about the role of APC in the development and/or peripheral expansion of Treg cells, nor about the mechanisms by which this occurs. It remains an attractive notion that by controlling the maturation of DCs, the outcome of an immune response can be modulated.

In order to mount an effective immune response in intestinal mucosa, the immune system has to discriminate harmful antigens on the one hand, and tolerate commensal bacterial and dietary antigens in the intestinal environment on the other hand. The failure to control these responses leads to a breakdown of tolerance towards resident microbiota and has been proposed as one mechanism involved in the development of inflammatory bowel diseases (IBD) [3]. It has also been shown recently that recognition of commensal bacteria by Toll like receptors (TLR) plays a crucial role in maintaining homeostasis [4]. Two recent reports have shown that the commensal flora could have an anti-inflammatory effect through an inhibition of the nuclear transcription factor NFκB, either by inhibition of IκB ubiquitination [5] or by regulating nuclear export of the NFκB relA subunit [6].

Probiotic bacteria, mainly belonging to the lactic acid bacteria (LAB) family, are well known to exert beneficial effects in human or animal health. Regular intake of probiotic bacteria contributes to immune homeostasis by altering microbial balance or by interacting with the gut immune system, explaining their potential effect in IBD. Consequently, therapeutic approaches designed to modulate the local microenvironment using probiotics, have been tested in animal models of colitis [7], as well as in human clinical trials with patients suffering from IBD [8]–[10]. Although there is a considerable body of information concerning the protective efficacy of probiotics, little is known about the precise mechanisms of action by which such bacteria may exert their beneficial effects. Interactions with TLRs and DCs in the gut are believed to be involved in this communication. It was recently reported that lactobacilli can differentially modulate DC maturation [11], [12]. Indeed, we and others have shown that probiotics may present distinct strain-specific immunomodulatory capacities both *in vitro* and *in vivo*. This strain-specific *in vitro* immunomodulation capacity seems to be closely correlated with the *in vivo* anti-inflammatory potential of the strain [13]. The anti-inflammatory effects of lactobacilli observed both after oral or systemic administrations [14], [15] suggest that mechanisms, distant from the site of inflammation could involve regulatory cell populations. Indeed, a recent *in vitro* study demonstrated that lactobacilli, in a strain-specific manner, were able to prime DCs to promote the development of Treg cells through DC-SIGN interaction [16]. Recent studies have shown that the conversion of DCs to tolerogenic DCs could lead to the inhibition of NFκB [17] and could be used therapeutically to prevent allograft rejection [18]. One of the mechanisms exploited by tolerogenic DCs involves expression of indoleamine 2, 3 dioxygenase (IDO), a tryptophan-catabolizing enzyme that can exert regulatory effects on T cells by tryptophan depletion [19], [20]. Local overexpression of IDO in IBD patients has been suggested as an anti-inflammatory mechanism to counterbalance the tissue-damaging effects of activated T cells infiltrating the colonic mucosa [21]. To our knowledge, tolerogenic DCs has never been used to protect against IBD. In a contribution to unravel the mechanism by which probiotics may exert their anti-inflammatory effects, we demonstrate in this paper that interaction of DCs with selected probiotic bacteria can lead to the induction of regulatory DCs able to protect mice from colitis

## Results

### LAB are efficiently taken up by BMDCs

**Figure 1:**
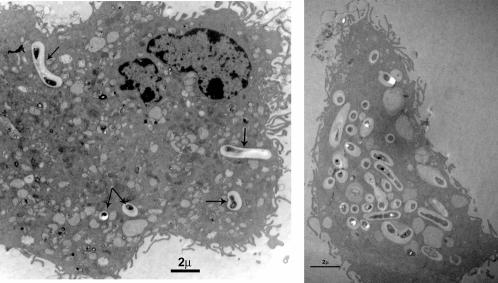
Internalization of *L. rhamnosus* by BMDCs. BMDCs were incubated for 18 h with Lr32 strain at a bacteria-to-DC ratio of 10 and then processed for transmission electron microscopy. Bacteria are visible within membrane-bound phagosomes at various stages of degradation (arrows). Bar, 2 µm.

We examined whether and how BMDCs internalized lactobacilli (*L. rhamnosus* Lr32 strain) by transmission electron microscopy. While after 2 h incubation at 37°C, only a small proportion of the bacteria was found inside the BMDCs (<10%) (data not shown), at 18 h most BDMCs (>80%) have internalized bacteria that show various stages of degradation. The bacteria were always included in vacuoles and phagosomes and never free in the cytosol (Fig. 1), suggesting that *L. rhamnosus* was taken up by conventional phagocytosis, as previously observed for *Streptococcus gordonii* with human DCs [22]. The number of bacteria present in each BMDC section ranged from 1 to 24 with an average of 7 bacteria per cell.

### Lactic acid bacteria differentially activate dendritic cells

**Figure 2:**
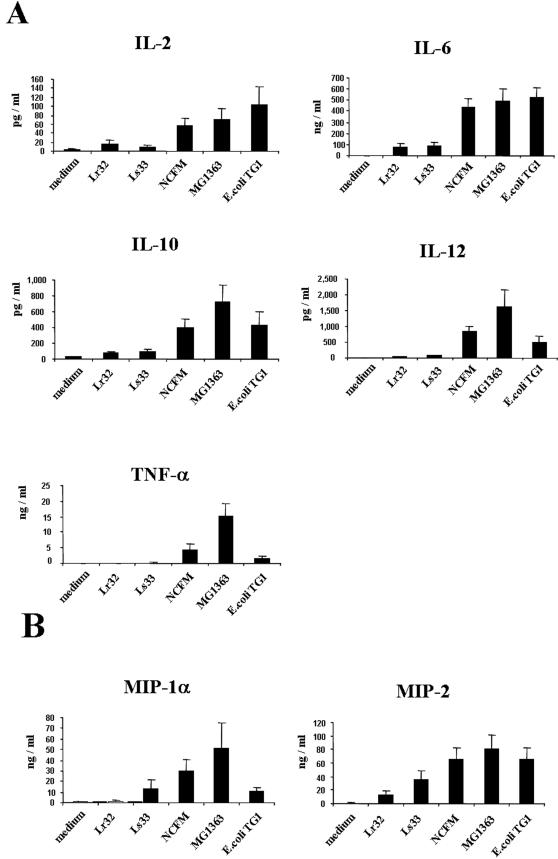
Cytokine (A) and chemokine (B) response of murine BMDCs (0.5×10^6^ cells/ml) derived from BALB/c mice to stimulation with strains *L. rhamnosus* Lr32, *L. salivarius* Ls3*3, L. acidophilus* NCFM, *L. lactis* MG1363 and *E. coli* TG1. Bacteria were collected after overnight culture and the stimulation of BMDC was done at a ratio of 10∶1 (bacteria/DC). Results represent the mean±SEM of 7 independent experiments.

We have previously observed that LAB have differential immuno-modulatory effects on PBMC, with some strains (i.e. *L. salivarius* Ls33, *L. rhamnosus* Lr32) having low *in vitro* pro-inflammatory and high immuno-regulatory capacities and correlating well with an efficient protective effect in murine colitis, and other strains (i.e. *L. acidophilus* NCFM, *L. lactis* MG1363) exhibiting an opposite immunological profile, and lacking a protective effect in vivo (Table 1). To determine the role of DCs in the anti-inflammatory capacity of these probiotic strains, we first evaluated the responses of mouse BMDCs to these LAB, using *E. coli* TG1 as a pro-inflammatory control strain. BMDCs were incubated with the bacteria at a ratio of 10∶1 in the presence of 150 µg/ml gentamycin, condition that completely impair bacterial overgrowth. Cytokine and chemokine productions were evaluated in the supernatant after 18–20 h culture (Fig. 2). The LAB strains NCFM and MG1363 as well as *E. coli* stimulated BMDC to produce large amounts of IL-6, TNF-α, MIP-1α and MIP-2 as well as significant levels of IL-12 and IL-10. In contrast, both Ls33 and Lr32 strains induced poor secretion of these cytokines and chemokines. Therefore, like observed in human PBMC model, LAB strains can be classified as pro-inflammatory and non-inflammatory also in the mouse BMDC infection model.

**Table 1:** Immuno-modulatory and protective properties of strains used in the study

Species	Strains	IL-10/IL-12^a^	protection^b^	Origine/Ref
*L. salivarius*	Ls33	36.2±5.8	+	commercial strain
*L. rhamnosus*	Lr32	15.75±5.07	+	commercial strain
*L. acidophilus*	NCFM	3.22±0.73	−	commercial strain
*L. lactis*	MG1363	0.78±0.18	−	cheese starter [53]
*E. coli*	TG1	31.82±7.26	−	commensal strain [54]

a IL-10/IL-12 ratio was previously determined by the capacity of the strains to induce IL-10 and IL-12 after *in vitro* PBMC stimulation and ^b^ protection indicated the capacity of the strains to protect mice from TNBS-induced colitis

**Figure 3:**
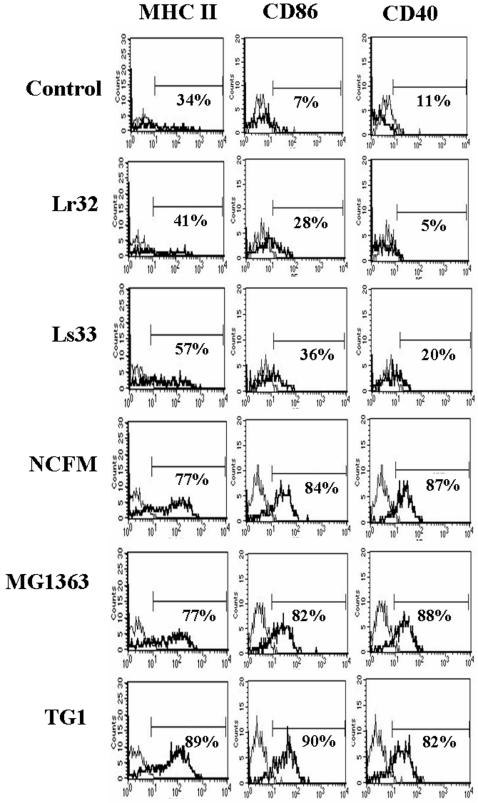
Phenotypic characteristics of non-treated (control) or bacteria-pulsed BMDCs from BALB/c mice. Plots show flow cytometric profiles of MHC class II, CD86 and CD40 expression (thick line) in comparison to an isotypic control (thin line). The data are representative of 4 independent experiments.

Next, we examined the ability of the LAB strains to activate co-stimulatory functions in BMDCs. Again a great difference was observed among the LAB strains in their capacity to up-regulate the expression of MHC class II and co-stimulatory molecules (CD86, CD40) (Fig. 3). Lr32 and Ls33, previously selected for their *in vitro* and *in vivo* anti-inflammatory properties, induced very low expression levels of co-stimulation markers as compared to untreated DCs, while strong and similar up-regulation was observed for the LAB strains NCFM, MG1363 and *E. coli*. Indeed, the two groups of LAB differ in their capacity to induce the production of pro-inflammatory cytokines by BMDCs and the surface expression of co-stimulatory signals. Subsets of DCs with such immature phenotype, called regulatory or tolerogenic DCs, are now recognized to induce and maintain peripheral tolerance. All these observations suggest that LAB strains Lr32 and Ls33 could exert their *in vivo* anti-inflammatory effects through induction of tolerogenic DCs.

### Administration of probiotic-treated BMDCs protect mice from colitis

**Figure 4:**
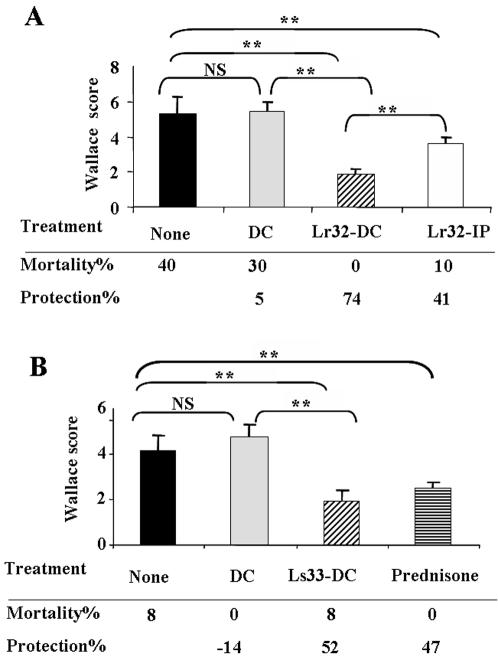
Protective effect of intra-peritoneal administration of LAB-treated BMDCs on acute TNBS-induced colitis in BALB/c mice. Wallace inflammation scores were calculated after a TNBS challenge in mice either not treated (None) or intraperitoneally injected by untreated BMDCs (DC) or BMDCs treated with (A) Lr32 (Lr32-DC) or (B) Ls33 (Ls33-DC) strains. The comparison between the TNBS-control groups and the groups that received the corresponding untreated BMDCs (DC) was calculated using the Mann-Whitney U test (<0.01, **; p<0.001, ***). Percentage mortality and protection (reduction of the mean Wallace scores of mice treated with BMDCs, in relation to the mean score of TNBS control group) are also indicated. In A, the effect of Lr32-treated BMDCs was also compared to the intra-peritoneal administration of 10^8^ CFU of Lr32 bacteria (Lr32-IP) and in B, an additional group of mice was included, pre-treated with 3 intra-peritoneal administrations of prednisone (10 mg/kg), representing a clinically relevant standard treatment for Crohn's disease (36). Data represent the mean±SEM of two representative experiments (number of mice n = 10).

**Figure 5:**
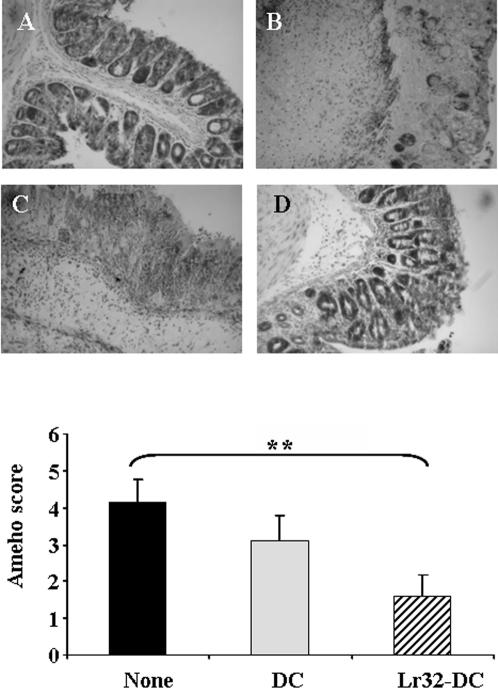
Representative histology of May-Grunwald stained sections (×10) of the distal colon from healthy control mice (A), mice with acute TNBS-induced colitis that have been or not (B) administered with non-treated DC (C) and Lr32-treated DC (D) and corresponding Ameho scores (E), mean±SEM (number of mice n = 10).

**Figure 6:**
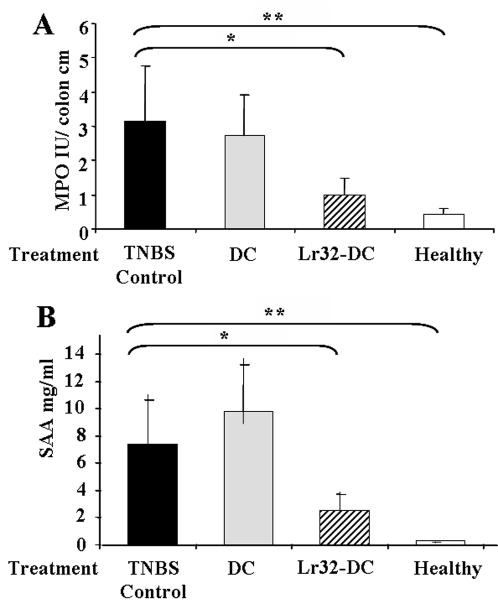
Colonic myeloperoxydase (MPO) activity (A) and Serum Amyloid A protein levels obtained 48 h after TNBS-induced colitis. Mice were left untreated (None) or IP administered with untreated BMDC (DC) or Lr32-treated BMDCs (Lr32-DC). The values are expressed as the mean±SEM (number of mice n = 10).

To investigate the role of DCs in the anti-inflammatory capacity of probiotic bacteria, we assessed whether the intraperitoneal administration of LAB-treated BMDCs could rescue mice from colitis induced by intra-rectal administration of TNBS. As compared to the TNBS-control group, the administration of untreated BMDCs did not rescue mice from colitis (p = 0.3116). In contrast, intra-peritoneal administration of *L. rhamnosus* Lr32 or *L. salivarius* Ls33-treated BMDCs, led to a considerable attenuation of the colitis, with reduced weight loss, improved clinical parameters (rectal bleeding, lethargy, and stool consistency; data not shown) and a significant reduction (p = 0.0003 and p = 0.0033, respectively) of macroscopic inflammation scores (Fig. 4A and 4B). Interestingly, the level of protection induced by probiotic-treated BMDCs was the same as that obtained with a pre-treatment by prednisone (at 10 mg/kg), a well known anti-inflammatory drug. Consistent with these data, the histological score of inflammation (Ameho score) was significantly reduced (p<0.01) in mice that were pre-treated with Lr32-DC, in comparison to the TNBS control group or the group of mice that received the non-treated DCs (Fig. 5E). We found substantial histological disruption of the villus and crypt structure concomitant with extensive cellular infiltration in the colonic lamina propria of mice that have been administered with TNBS alone (Fig. 5B) or with non-treated DCs (Fig. 5C). As expected, colonic inflammation, induced by TNBS, was totally abolished in mice that were transferred with Lr32-treated DCs (Fig. 5D), resulting in almost similar pictures as observed in healthy control mice (Fig. 5A). Additionally, the degree of polymorphonuclear neutrophil infiltration, assessed by quantifying myeloperoxidase (MPO) activity in colon extracts was also significantly decreased (p<0.05) in mice that received the Lr32-treated DCs in comparison to the TNBS control group (Fig 6A). Similarly, a decrease of SAA was also obtained in the sera of mice that had been treated with Lr32-pulsed DCs (Fig 6B). The protective effect of Lr32-treated BMDCs was verified in three additional experiments, leading to 59 to 74% protection (data not shown). Moreover, we confirmed as previously published [15] that intra-peritoneal administration of 10^8^ CFU of Lr32 bacteria could also significantly (p = 0.01) protect mice from colitis, where, interestingly, the protection induced by Lr32-treated BMDCs was found to be significantly (p = 0.0036) higher (Fig 3A). Finally, we evaluated in two independent experiments, the effect of Lr32-pulsed DCs in an acute DSS-induced model of colitis (7 days of daily consumption of drinking water containing 6% DSS). This caused loose and bloody stools (9/9 mice), progressive weight loss and significant shortening of the colon in control mice. However, when mice were pre-treated with Lr32-pulsed DCs, we only partially abolished the bloody stools (6/9), but we measured no significant amelioration of weight loss, colon size or inflammation parameters (MPO) (data not shown).

### The protective effect of probiotic-treated BMDC is associated with a down-regulation of pro-inflammatory mediators

**Figure 7:**
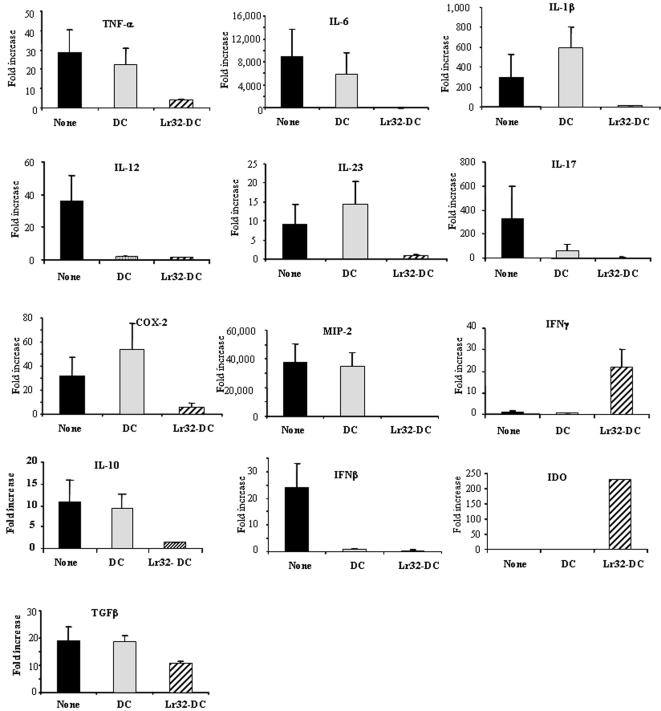
Quantitative real time PCR analysis of mRNA expression of pro-inflammatory or regulatory mediators in colons obtained 48 h after TNBS-induced colitis. Mice were left untreated (None) or IP administered with untreated BMDC (DC) or Lr32-treated BMDCs (Lr32-DC). The values are expressed as the mean ratio±SEM of mRNA levels after TNBS challenge compared with non challenged healthy mice.

In order to analyze the mechanisms of protection of probiotic-pulsed BMDCs in TNBS-induced colitis, the modulation of the expression of selected genes was assessed in the animal colons by real time PCR using the Taq-Man qRT-PCR technology. As shown in Figure 7, the colitis, developed after TNBS administration, largely enhanced the expression of TNF-α, IL-6, IL-1β, IL-12, IL-23, IL-17, Cox-2, IL-10, and IFNβ genes, with a fold increase in mRNA levels of 28.7, 89.26, 303.9, 36.03, 9.14, 329.00, 32.07, 10.89, and 24.16, respectively compared to healthy mice. A dramatic increase of MIP-2 gene expression was also observed showing a 37,626 fold increase compared to naïve mice. Interestingly, by comparing individually the Wallace inflammation scores and the gene expression levels, an excellent correlation was found using the Spearman rank test, (*r* values ranging from 0.75 to 0.84; data not shown). The administration of untreated BMDCs had no significant impact on the global expression of pro-inflammatory genes. However, we observed a slight upregulation of IL-1β, IL-23 and COX-2 expression, while a downregulation of IL-12 and IL-17 expression was detected. As expected, administration of Lr32-treated BMDCs greatly reduced the expression of all these genes. Surprisingly, IL-10 and IFNβ expressions were also up-regulated during colitis induction and were down-regulated upon probiotic-pulsed DC treatment. In addition, the expression of IFNγ and IDO was not altered in the colon upon colitis induction. In contrast, treatment of animals with Lr32-pulsed DCs promoted an acute over-expression of IFNγ and IDO (Fig 7). Finally, FoxP3 colonic expression was not modulated in any group of mice (data not shown).

### Depletion of CD4+ CD25+ cells totally abrogated the protective effect induced by probiotic-treated DC

**Figure 8:**
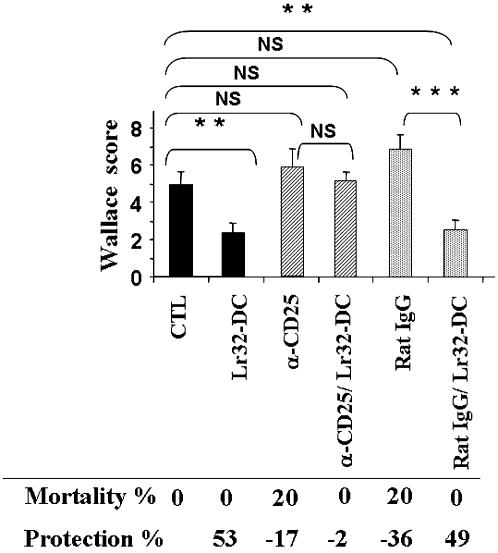
Result of the *in vivo* administration of anti-CD25 mAb (PC61) on the protective effect of probiotic-pulsed BMDCs in TNBS-induced colitis. Ten mice per group were challenged with TNBS alone (CTL) or after Lr32-pulsed BMDC transfer (Lr32-DC). The effect of a pre-treatment by either 200 µg of anti-CD25 (α-CD25) or control rat IgG (rat IgG) mAb, 24 h before colitis induction was analyzed in mice treated or not by Lr32-pulsed BMDC (Lr32-DC) transfer. Wallace inflammation scores (mean ± SEM) were calculated in each group and compared to each other by a Mann-Whitney U test (<0.01, **; p<0.001, ***). Percent of mortality and protection (reduction of the mean Wallace scores of mice treated with BMDCs, in relation to the mean score of TNBS control group) are also reported.

Previous studies have shown that anti-CD25 monoclonal antibody (mAb) was capable of depleting CD25^+^ Treg cells *in vivo* [23] with a loss of CD4^+^ FOXP3^+^ cells as recently commented by Zelenay and Demengeot [24] and by Stephens and Anderton [25]. To analyze the involvement of the naturally occurring CD4+ CD25+ T cell population in the protective effect of probiotic-treated BMDCs, we investigated the effect of *in vivo* intra-peritoneal administration of an anti-CD25 rat mAb in comparison to the effect of an isotype control rat IgG. Depletion was confirmed 24 and 48 h after injection in spleen and mesenteric lymph nodes, by flow cytometry using anti FITC-labeled anti-CD4 and PE-labeled anti-CD25 mAbs (data not shown). As shown in Figure 8, similar inflammatory scores were obtained in TNBS, anti-CD25/TNBS- and isotype/TNBS-treated groups, confirming that the injection of both mAbs had no significant impact on the TNBS-induced colitis (p = 0.25 and 0.20, respectively). As expected, Lr32-treated BMDCs significantly protected mice against TNBS-induced colitis in untreated mice (p = 0.004) or in mice injected with irrelevant rat IgG (p = 0.004). In contrast, depletion of CD25+ cells totally abrogated the protective effect of probiotic-treated BMDCs (p = 0.45). These experiments suggest that probiotic treatment of DC promotes *in vivo* a protective effect dependent on the CD4+ CD25+ Treg population.

### PRR signaling is required for anti-inflammatory effects of probiotic-treated BMDCs

To analyse the requirement of pattern recognition receptors (PRR) signaling in the anti-inflammatory effects of probiotic-treated DCs, BMDCs were derived from C57BL/6 or TLR2^−/−^, MyD88^−/−^ and NOD2^−/−^ KO mice. We also analyzed the role of IL-10 by using BMDCs derived from IL-10^−/−^ mice. The defect in TLR2 and NOD2 in the respective KO mice used was confirmed using specific ligands, Pam3Cys-Ser-(Lys)4 (Pam3C) and N-Acethylmuramyl-L-alanyl-D-iso-glutamine hydrate (MDP) respectively, using lipopolysaccharide (LPS) as positive control (data not shown). The capacity of LAB to activate BMDCs derived from C57/Bl6 (the genetic background of deficient mice) was similar to results obtained in BALB/c (Figure 1 and 2). No major differences were observed in the activation of BMDCs derived from TLR2- and NOD2-deficient mice. Stimulation of cells derived from the IL-10^−/−^ mice, also had no effect other than the expected absence of IL-10 production. In contrast in MyD88^−/−^ DCs, cytokine and chemokine production was totally abolished and a partial reduction of co-stimulation was observed, as previously reported [26] (data not shown).

**Figure 9:**
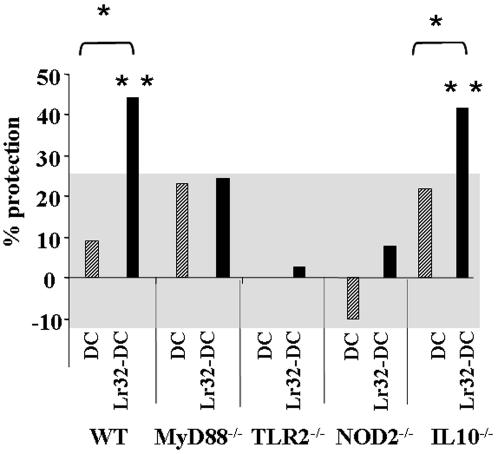
Protective effect of intra-peritoneal administration of untreated (DC) or Lr32-treated BMDCs (Lr32-DC) derived from C57Bl/6 WT or MyD88^−/−^, TLR2^−/−^, NOD2 ^−/−^ or IL-10^−/−^ KO mice on TNBS-induced colitis in C57Bl/6 mice. For technical reason, the protective effect of all KO BMDC's have not been analyzed in the same experiment. For that reason, results are only expressed as the reduction (in %) of the mean macroscopic inflammation scores of mice treated with BMDCs, in relation to the mean scores of non-treated mice (corresponding control TNBS). Significance values p<0.05 (*) or p<0.01 (**), as compared to the corresponding TNBS-control group or the groups that received the corresponding non-treated BMDCs, were calculated by the Mann-Whitney U test (n = 10). The dashed line indicates the 25% threshold of the uncertain statistical significance as previously described (48).

We finally evaluated whether the administration of Lr32-treated BMDCs derived from KO mice could protect C57BL/6 mice from colitis. As C57BL/6 are more resistant to TNBS-induced colitis [27], a higher dose of the hapten was used to obtain a similar score of inflammation. Under these comparable conditions, Lr32-treated BMDCs from C57BL/6 mice again induced a significant protection when compared to the TNBS control group (p = 0.01) and the group that received the untreated BMDCs (p = 0.03) (Fig. 9). Similar results were obtained when using BMDCs derived from the IL-10^−/−^ mice, indicating that IL-10 was not involved in the Lr32-mediated protection. In the same conditions, the protective effect of Lr32-treated DCs was completely abolished when cells were derived from MyD88^−/−^ , TLR2^−/−^ and NOD2^−/−^ mice (non-significant p-values of 0.42, 0.38, 0.11, respectively, versus the TNBS, non-treated WT mice), suggesting that the anti-inflammatory effects conferred by Lr32-treated DC required signaling involving these molecules. We could therefore conclude that the protective effect against colitis likely requires innate detection of microbial products derived from specific LAB strains by DCs, thereby leading to the development of tolerogenic DC dependent on active cell signalling.

## Discussion

It is now well accepted that in genetically susceptible individuals, intestinal microbes can contribute to the initiation and perpetuation of chronic mucosal inflammation. Various factors are likely involved in the loss of tolerance to the patient's commensal flora in IBD. Although many types of immune cells regulate immune responses, most attention has focused on regulatory T cells as a way to cure or prevent colitis in mouse models of intestinal inflammation [28]. Partially mature DCs possess inherent tolerogenic properties, and it is an attractive notion that by controlling or directing the maturation of DCs, T cell activation can be biased towards the generation of Treg cells. This principle, when managed properly, could proof to be extremely valuable in correcting, in a controlled way, a large number of immune disorders. Preventive properties of probiotic microorganisms against colonic inflammation have been reported [8]. Nevertheless, the unraveling of the mechanism of action is mandatory to allow the establishment of criteria for the selection of the most efficient strains. Immunoregulatory effects of probiotic strains seem to be strain-specific and linked to the composition of the cell surface [29]. We and others previously showed that probiotics are able to exert anti-inflammatory effects distant from the site of administration, suggesting the involvement and migration of regulatory cells [14], [15]. Two recent studies reported that probiotics can improve murine colitis by inducing TGF-β bearing regulatory T cells [30] and can induce *in vivo* peripheral T cell hypo-responsiveness [31], suggesting a modulation through DC function. In the present study, we particularly addressed the role of DCs in the protective effect mediated by probiotic strains. We confirmed that LAB differentially interact with DCs. The strains *L. rhamnosus* Lr32 and *L. salivarius* Ls33, while being taken up very efficiently by DCs, were unable to trigger cytokine and chemokine production as well as the expression of co-stimulatory molecules, in contrast to other LAB strains investigated. We hypothesized therefore that Lr32 and Ls33 induced the differentiation of DCs into tolerogenic DCs. Indeed, the probiotic-pulsed DCs exhibiting this tolerogenic phenotype, were able to protect mice against TNBS-induced colitis, after intra-peritoneal administration. The tolerogenic properties of DCs are not only associated with a failure to mature since untreated immature DCs are unable to confer protection. This protective effect by tolerogenic DC's is probably conveyed by an effect of T cells. According to previous studies, immune mechanisms involved in the inflammatory processes are different between DSS- and TNBS-induced colitis [32]. While macrophages seem to be involved in the DSS model, T cells play a pivotal role in the TNBS-mediated colitis. In this study, we could not find any substantial effect of probiotic-pulsed DCs on DSS-induced colitis, suggesting that the treatment does not regulate macrophage functions in the pathogenesis of colitis. Similar observations have been reported for several treatments that protected in one model but not in the other [33], [34]. TNBS colitis is associated with a Th1-mediated immune response, which clearly mimics human IBD. Therefore the enhancement of transcription of pro-inflammatory cytokine genes has been reported in this model [35]. We also observed that the expression of pro-inflammatory mediators was strongly up-regulated in TNBS-induced colitis while strongly reduced in mice treated with Lr32-pulsed DCs. It was established that IL-23 plays a key role in IBD by driving the development of IL-17 producing T cells that promote the production of the main inflammatory mediators [36]. We could show increased mRNA expression levels of both IL-23 and IL-17 upon TNBS treatment, which were greatly reduced by Lr32-pulsed DCs. As previously reported [37], TNBS also strongly induced mRNA expression for IL-10, which could not be inhibited by potent anti-inflammatory drugs. In the present study, we observed a similar increase of IL-10 mRNA levels as well as TGFβ after TNBS administration which was prevented by Lr32-pulsed DCs. Surprisingly, while the two protective strains used in this study were previously shown to induce high IL-10 production in human PBMCs (Table 1), we could not confirm this property when using murine BMDCs. Nevertheless, in both *in vitro* models, low production of pro-inflammatory mediators was an appropriate indicator of strains harboring protective efficacy in colitis. IL-10-dependent regulatory cells have been suggested to be involved in the protective effects of probiotics. However, in IL-10 deficient mice, which spontaneously develop colitis, probiotic administration could resolve inflammation, suggesting that their protective effect could be IL-10 independent [7]. In our study, mice were indeed protected by Lr32-pulsed BMDCs derived from IL-10 deficient mice, suggesting that the beneficial effect elicited by conditioned DCs is also independent of IL-10.

Moreover, we could demonstrate that the protective effect of probiotic-conditioned BMDCs was linked to the presence of CD4+ CD25+ cells, since protection was not achieved in mice depleted of CD25+ cells. Similarly, it has been shown that immature DCs, whose NFκB-signaling pathway was blocked by a proteosome inhibitor, could be a source of antigen-specific regulatory cells that displayed *in vitro* and *in vivo* inhibitory effects in an IL-10 independent manner [38]. The mechanisms, by which these regulatory T cells are derived, remain to be defined. Grohman [19] proposed that tolerogenic DCs could exert regulatory effects on T cells by the expression of indoleamine 2, 3 dioxygenase (IDO), that lead by locally depleting tryptophan to initiates an immunosuppressive pathway. The colonic over-expression of IDO we observed, suggests that it could be one mechanism by which probiotic-treated DCs could exert regulatory effects. Interestingly, in the colon of protected mice we observed a parallel enhancement of IFNγ, which has been shown to be the principal inducer of IDO expression.

The innate immune system is triggered by various pattern recognition receptors (PRR), including TLR and NOD families. PRR signaling must be properly regulated and multiple pathways seem necessary to dampen or reduce the signaling, thereby maintaining immune homeostasis. While deregulated interaction between molecular pattern and TLRs may promote chronic inflammation and tissue damage, recognition of commensal bacteria by TLRs plays an important role in maintaining intestinal homeostasis and contributes to the prevention of intestinal injury [4]. To investigate the role of PRR signaling in the protective effects of probiotic, Lr32-pulsed BMDCs derived from MyD88, TLR2 and NOD2 deficient mice were transferred to TNBS-treated mice. The administration of deficient BMDCs conditioned by the probiotic strain, did not affect the course and severity of colitis, indicating the involvement of both the TLR2 and NOD2 signaling pathways in the control of the colitis. Since the innate immune system of the intestine has evolved in the presence of luminal bacteria, it is reasonable to hypothesize that normal intestinal function may be regulated by bacteria through TLRs. CpG oligonucleotides have also been shown to prevent murine colitis, supporting also the essential role of TLR9 signaling [39]. Genetic evidence supports a role of PRR in IBD, since polymorphisms in NOD2 and to a lower extent in TLR9 genes have been linked with Crohn's disease [40], [41]. Recently Watanabe and colleagues proposed that peptidoglycan (PGN)-mediated activation of TLR2 signaling is negatively regulated by MDP-mediated activation of NOD2 signaling [42]. In the absence of NOD2, the negative regulation in response to PGN is impaired, leading notably to an enhanced IL-12 production both *in vitro* and *in vivo*. The observed synergy between TLR9 and NOD2 is apparently lacking in NOD2 homozygous CD patients, suggesting implications for PRR-mediated intestinal homeostasis and inflammation [43]. Whatever the details of the mechanisms involved, it is now apparent that the function of TLRs and NODs could be combinatorial and might be synergistic or antagonistic and that PRR signaling is likely to promote the induction of regulatory cells to maintain tissue homeostasis.

We recently illustrated the importance of cell wall components in the pro- versus anti-inflammatory properties of LAB [29]. As suggested by Travasso *et al* [44], we could speculate that PGN from tolerogenic probiotic could impact on the TLR2 signaling cascade through NOD2 interaction. Nevertheless, we could not exclude the potential role of DNA mediated by TLR9 signaling in the effect of probiotics on DC function. These results raise the critical question of how certain bacteria exert (or not) their anti-inflammatory potential. To answer this question, purification of bacterial components of different probiotic strains, able (or not) to prime tolerogenic DCs is in progress. By analyzing both *in vitro* and *in vivo* effects of such compounds, we hope to unravel exactly how probiotics exert their immunoregulatory effects, and clarify the role of PRR signaling.

Finally, perspectives for the use of probiotic-primed DCs are manifold. Substantial reports describe immunosuppressive agents, able to generate regulatory DCs that can be used to treat transplant rejection and autoimmune diseases [45], but nothing has been published so far for IBD. Despite increasing knowledge about the immune-pathology of IBD, the therapeutic options are still limited today. In this work we demonstrated that probiotics, however, are able to induce tolerogenic DCs, offering a potential therapy and providing important clues as to the mechanisms underlying their anti-inflammatory properties. The selection of suitable probiotic strains, bacterial components or the design of potent cell wall mutants or recombinant strains to condition DCs may become an attractive alternative for treating IBD or other immunological disorders.

## Materials and Methods

### Bacterial strains and growth conditions

Lactic acid bacteria used in this study are listed in Table 1. Lactobacilli were grown at 37°C in MRS broth (Difco, Detroit, Mich.), *L. lactis* strain was grown at 30°C in M17 supplemented with 0.5% glucose and the *E. coli* TG1 strain was grown at 37°C in Luria broth. For cell stimulation, bacteria were grown overnight, harvested by centrifugation, washed twice in sterile PBS pH 7.2, and resuspended at 10^9^ CFU per ml in PBS containing 20% glycerol. Suspensions were stored at −80°C until used for stimulation assays. For direct intra-peritoneal administration to mice, bacteria were grown overnight, washed twice and resuspended at 10^8^ CFU/ml in PBS.

### Bone Marrow-Dendritic Cell (BMDC) generation

BMDCs were generated from the bone marrow precursors isolated from femurs and tibias of mice as described by Lutz *et al*. [46], with minor modifications. Bone marrow cells were prepared from wild-type female BALB/c or C57BL/6 mice (Charles river, France) and from TLR2 knock-out (KO) mice (TLR2^−/−^) (obtained from S. Akira [47] (Osaka University, Osaka, Japan), via a generous gift of B. Ryffel (CNRS GEM2358, Orleans, France) and F. Trottein (INSERM U567, Lille, France), MyD88 KO mice (MyD88^−/−^) [47], IL-10 KO (IL10^−/−^) mice (Charles river, France). C57BL/6 background *NOD2* KO mice were generated in the laboratory of M. Giovannini (Barreau et al, in preparation). The first *NOD2* coding exon carrying the majority of the sequence encoding the CARD domains including the start codon, was deleted resulting in a null *NOD2* allele. Germ line transmitting chimeras were crossed to C57BL/6 mice to generate NOD2^+/−^ mice. After ten backcrosses with C57BL/6 mice, *NOD2* ^+/−^ mice were intercrossed to generate NOD2^−/−^ mice. NOD2^−/−^ mice were indistinguishable from controls and showed no signs of liver pathology. All mice were housed in specific pathogen free (SPF) conditions. The defect in TLR2 and NOD2 in the respective KO mice was confirmed using specific ligands, respectively Pam3Cys-Ser-(Lys)4 (PamC3, at 15 µg per ml, Calbiochem, Germany) and N-Acethylmuramyl-L-alanyl-D-iso-glutamine hydrate (MDP, at 100 µg per ml, Sigma, ST-Louis, MI, USA) while LPS (at 100 ng per ml, Sigma) was used as positive control (data not shown). Briefly, after red cell lysis, BM cells were cultured at 2×10^5^ cells/ml using Petri dishes and Iscove medium (Sigma-Aldrich, St. Louis, MO) supplemented with 10% heat-inactivated fetal calf serum (FCS, Gibco-BRL, Paisley, Scotland), 50 µM 2-mercaptoethanol, 1 mM glutamine, 50 µg/ml gentamycin and 10% of supernatant from a granulocyte-macrophage colony-stimulating factor-expressing cell line (GM-CSF transfected J588 myeloma cell line). Freshly prepared medium was added every three days and BMDCs were used on day 11 of culture (maximum of CD11c expression as checked by FACS analysis).

### BMDC stimulation

BMDCs (5×10^5^ cells/ml) were stimulated with 5×10^6^ bacteria/ml in the presence of 150 µg/ml gentamycin or were left untreated. After 18–20 h, culture supernatants were collected, clarified by centrifugation and stored at −20°C for cytokine and chemokine analysis. For *in vivo* cell transfer, BMDCs were washed 3 times in PBS and resuspended at 2×10^6^ cells in 200 µl. No remaining viable bacteria were detected in the cell suspensions, as checked by plating on MRS medium. Conditioned medium was obtained by using the supernatant after filtration using a 0.22 µM Millipore™ membrane (Bedford, MA).

### Electron microscopy

LAB-pulsed BMDC were processed for electron microscopy according to Noël et al [48], slightly modified. Briefly, after the primary fixation, cells were washed 3 times in 0.1 M sodium cacodylate buffer pH 7. 4, pellets were encapsulated in 2% solution of low melting point agarose, before proceeding to post-fixation, dehydration and embedding. Ultrathin sections were stained with uranyl acetate, lead citrate and examined under a Hitachi 7500 electron microscope.

### Immunocytostaining and flow cytometry

For FACS analysis (expression of surface markers), cells were collected by gentle pipetting, centrifuged for 10 min at 300× g, and resuspended in cold PBS containing 1% (v/v) FCS, and 0.1% sodium azide (PBS-FCS-Az). For all staining, cells were incubated with Fc receptor-blocking mAb anti-CD32 (2.4G2). The following Abs used for staining were purchased from BD Pharmingen: FITC-conjugated anti-mouse CD11c (HL3); PE-conjugated anti-mouse MHC II (I-A/I-E; M5); PE-conjugated anti-mouse CD86 (GL1); PE-conjugated anti-mouse CD80 (16-10A1); PE-conjugated anti-mouse CD40 (3/23) and appropriate mAb isotypic controls and from eBioscience: FITC-conjugated anti-CD4 (RM4-5) and PE-conjugated anti-CD25 (PC61.5). Cells were incubated with selected mAb for 30 min on ice and at low light exposure. Thereafter, cells were washed with 3 ml PBS-FBS-Az and finally resuspended in 300 µl paraformaldehyde 1% for flow cytometric analysis using a FACS-Calibur flow cytometer and CellQuest software (BD Biosciences, San Jose, CA).

### Cytokine and chemokine quantification in culture supernatants

Murine IL-12(p70), IL-10, IL-6 and TNF-α were analyzed using matched Ab pairs purchased from BD Pharmingen (BD Biosciences, San Jose, CA). MIP-1α and MIP-2 were similarly analyzed using commercially available ELISA kits (R&D systems, Minneapolis, MN) according to the manufacturer's instructions.

### Induction of colitis

Animal experiments were performed in an accredited establishment (number A59107; animal facility of the Institut Pasteur de Lille, France) and carried out in accordance with the guidelines of laboratory animal care published by the French Ethical Committee and the rules of the European Union Normatives (number 86/609/EEC). BALB/c and C57Bl/6 (female, 8 weeks) were obtained from Charles River (St Germain sur l'Arbresle, France). A standardized murine TNBS colitis model was used to induce acute levels of inflammation [49]. Briefly, anesthetized mice received an intra-rectal administration of 50 µl solution of 2,4,6-trinitrobenzene sulfonic acid (TNBS, Sigma-Aldrich Chemical, France) (100 mg/kg for BALB/c mice and 150 mg/kg for C57Bl6 mice) dissolved in 0.9% NaCl/ethanol (50/50 v/v). Colons were removed at sacrifice, 48–72 h after administration, washed and opened. Inflammation grading was performed blindly using the Wallace scoring method [50], reflecting both the intensity of inflammation and the extent of the lesions. The protective effect of untreated or LAB-pulsed DCs was studied by intra-peritoneal administration of 2×10^6^ cells, 2 h before TNBS-colitis induction. The same protocol was performed using 10^8^ CFU live bacteria, administered intra-peritoneally before colitis induction. A commercial preparation of prednisone (Cortancyl, Sanofi Aventis, France) was used as positive control of protection and was orally administered for 3 subsequent days at 10 mg/kg starting at the day of TNBS administration. Histological analysis was performed on May-Grünwald-Giemsa stained 5 µm tissue sections from colon samples fixed in 10% formalin and embedded in paraffin. The myeloperoxidase (MPO) activity was determined in distal colon tissue by specific kinetic assays according to Bradley et al [51]. Immediately after sacrifice, colonic samples were taken and snap-frozen on liquid nitrogen after which they were stored at −80°C. Pure MPO from human neutrophils (Sigma-Aldrich Chemical, France) was used as a standard. One unit of MPO activity was defined as the quantity of MPO degrading 1 μmol hydrogen peroxide/min/ml at 25°C. Data are expressed as international unit (IU) of MPO per cm of colon tissue. Serum amyloid A (SAA) protein were measured by ELISA using commercial kits from Biosource International (Camarillo, Ca, USA) according to the manufacturer recommendations.

For RT-PCR analysis, a part of the colon corresponding to the enflamed region was cleaned from fecal material and stored in RNA-later^R^ buffer (Ambion, Austin, TX, USA) at −80°C until RNA extraction.

### In vivo depletion of CD4+ CD25+ population

In order to analyze the impact of the CD4+ CD25+ Treg cell population, CD25+ cells were depleted by intra-peritoneal injection of 200 µg per BALB/c mice of purified rat IgG monoclonal antibody (mAb) anti-CD25 produced by hybridoma PC61 [52], in comparison with an isotypic control (mAb ATCC HB 152), 24 h before BMDCs administration and TNBS-colitis induction. Depletion was checked by flow cytometry using anti FITC-labeled anti-CD4 and PE-labeled anti-CD25 mAbs.

### Quantitative real time RT-PCR

Total RNA from individual colons was purified by using NucleoSpin RNA II kit (Macherey-Nagel, Düren, Germany), including a DNase treatment according to the manufacturer's recommendations. Reverse transcription of total RNA (2 µg) was performed with random primers (Amersham, Piscataway, NJ, USA) and Superscript II (Invitrogen, Carlsbad, CA, USA). Fifty nanograms of template were used in a real-time PCR reaction with a final volume of 25 µl using the Taq-Man PCR Master Mix (Applied Biosystems, Branchburg, NJ, USA), and the primers and probes designed by Applied-Biosystems (Assays-on-demand) for murine TNF-α (Mn00443258), IL-6 (Mm00446190), IL-1β (Mm00434228), IL-12 (Mm00434170), IL-23 (Mn00518984), IL-17 (Mm00439619), Cox-2/Ptgs2 (Mn00478374), MIP-2/CxCl2 (Mn00436450), IFNγ (Mm00801778), IL-10 (Mn00439616), IFNβ (Mm00439546), TGFβ (Mm004441724), IDO (Mm00492586) and β-actin (Mn00607939), as suggested by the manufacturer. All reactions were performed in duplicate, and the thermal cycling conditions were 10 min at 95°C, followed by 40 cycles of 15 s at 95°C and 1 min at 60°C using the Applied Biosystems 7300 real time PCR system. For relative quantification, we compared the amount of target normalized to the β-actin amplification by using the 2 ^−ΔΔCt^ formula representing the n-fold differential expression of the target gene in the treated sample, compared with sample from naïve mice (control), where Ct is the mean of threshold cycle, ΔCt was the difference in the Ct values for the target gene and the β-actin reference gene (for each sample), and ΔΔCt represents the difference between the Ct from the control and each sample. Non-template (NTC) and Reverse-transcription (RTC) control were included for each quantification experiment.

### Statistical analysis

Comparisons between the different animal groups were analyzed by the non-parametric one–way analysis of variance, Mann-Whitney U test. Differences were considered to be statistically significant when the p value was <0.05. For *in vivo* experiments, only protection levels exceeding the 25% level (positive and negative) were considered to be relevant, as previously shown [48].
